# Evaluation of strategies to support implementation of a hospital walking program: protocol for a type III effectiveness-implementation hybrid trial

**DOI:** 10.1186/s43058-024-00544-5

**Published:** 2024-01-12

**Authors:** Caitlin B. Kappler, Cynthia J. Coffman, Karen M. Stechuchak, Ashley Choate, Cassie Meyer, Leah L. Zullig, Jaime M. Hughes, Connor Drake, Nina R. Sperber, Brystana G. Kaufman, Courtney H. Van Houtven, Kelli D. Allen, Susan N. Hastings

**Affiliations:** 1 Center of Innovation to Accelerate Discovery and Practice Transformation, Durham VA Health Care System (152), 508 Fulton Street, Durham, NC 27705 USA; 2 Department of Biostatistics and Bioinformatics, Duke University School of Medicine, Durham, NC USA; 3 Department of Population Health Sciences, Duke University School of Medicine, Durham, NC USA; 4 Department of Implementation Science, Wake Forest School of Medicine, Winston-Salem, NC USA; 5 Section On Gerontology and Geriatric Medicine, Department of Internal Medicine, Wake Forest School of Medicine, Winston-Salem, NC USA; 6 Duke-Margolis Center for Health Policy, Duke University, Durham, NC USA; 7 Department of Medicine & Thurston Arthritis Research Center, University of North Carolina at Chapel Hill, Chapel Hill, NC USA; 8 Center for the Study of Aging and Human Development, Duke University School of Medicine, Durham, NC USA; 9 Geriatrics Research, Education, and Clinical Center, Durham VA Health Care System, Durham, NC USA; 10 Department of Medicine, Division of Geriatrics, Duke University School of Medicine, Durham, NC USA

**Keywords:** Function/mobility, Implementation science, Veterans, Supervised walking, Aging

## Abstract

**Background:**

STRIDE is a supervised walking program designed to address the negative consequences of immobility during hospitalization for older adults. In an 8-hospital stepped wedge randomized controlled trial, STRIDE was associated with reduced odds of hospital discharge to skilled nursing facility. STRIDE has the potential to become a system-wide approach to address hospital-associated disability in Veteran’s Affairs; however, critical questions remain about how best to scale and sustain the program. The overall study goal is to compare the impact of two strategies on STRIDE program penetration (primary), fidelity, and adoption implementation outcomes.

**Methods:**

Replicating Effective Programs will be used as a framework underlying all implementation support activities. In a parallel, cluster randomized trial, we will use stratified blocked randomization to assign hospitals (*n* = 32) to either foundational support, comprised of standard, low-touch activities, or enhanced support, which includes the addition of tailored, high-touch activities if hospitals do not meet STRIDE program benchmarks at 6 and 8 months following start date. All hospitals begin with foundational support for 6 months until randomization occurs. The primary outcome is implementation penetration defined as the proportion of eligible hospitalizations with ≥ 1 STRIDE walks at 10 months. Secondary outcomes are fidelity and adoption with all implementation outcomes additionally examined at 13 and 16 months. Fidelity will be assessed for STRIDE hospitalizations as the percentage of eligible hospital days with “full dose” of the program, defined as two or more documented walks or one walk for more than 5 min. Program adoption is a binary outcome defined as ≥ 5 patients with a STRIDE walk or not. Analyses will also include patient-level effectiveness outcomes (e.g., discharge to nursing home, length of stay) and staffing and labor costs. We will employ a convergent mixed-methods approach to explore and understand pre-implementation contextual factors related to differences in hospital-level adoption.

**Discussion:**

Our study results will dually inform best practices for promoting successful implementation of an evidence-based hospital-based walking program. This information may support other programs by advancing our understanding of how to apply and scale-up national implementation strategies.

**Trial registration:**

This study was registered on June 1, 2021, at ClinicalTrials.gov (identifier NCT04868656).

Contributions to the literature

- This study will contribute to a better understanding of how to implement hospital walking programs in real-world clinical settings.
- This study will improve the efficiency and impact of large-scale implementation within learning health systems.
- Results will provide insight to implementing walking programs not only in the Veteran’s Affairs but could potentially translate to healthcare systems across the USA.

## Background

Immobility plays a central role in causing hospital-associated disability, impacting over one-third of adults aged 70 and above who experience a significant new disability upon discharge that was not present before hospital admission [1]. Despite physician orders for bedrest being less than 5%, older adults in hospitals only spend around three percent of their time engaged in standing or walking. The dangers of prolonged bedrest have been acknowledged for decades, yet an epidemic of immobility persists in hospitals [2]. This results in increased discharges to post-acute care facilities, which are intended for short-term rehabilitation but carry a substantial risk of long-term institutionalization upon admission [3]. Despite ample evidence highlighting the negative impact on patients and costs to the healthcare system, there are still gaps in clinical practices aimed at promoting mobility in hospitals [4–6].

Hospitals are implementing walking programs as a strategy to mitigate functional decline in older adults during their hospital stay. Previous studies have demonstrated that daily ambulation can enhance function and walking ability upon discharge, as well as prevent the loss of community mobility 1 month after leaving the hospital [7]. When hospitals aim to successfully introduce new clinical programs, particularly those requiring coordination among multiple providers and changes in workflow, active implementation support is often necessary [8]. Nevertheless, there remains a knowledge gap regarding the most effective methods to address variation in hospital needs when implementing programs on a large scale in diverse settings.

## Methods and design

STRIDE (Assi*ST*ed Ea*R*ly Mob*I*lity for Hospitalize*D* V*E*terans) is an evidence-based program (EBP) within the Optimizing Function and Independence Quality Enhancement Research Initiative (Function QUERI), aimed at enhancing the functional independence of older Veterans. Several hospitals within the Veterans Affairs (VA) system showed interest in initiating STRIDE walking programs. This provided an opportunity to gather evidence on the impacts of this program and study two strategies for assisting hospitals in implementing STRIDE.

### Study goals and objectives

The current protocol builds upon work that began with initial funding in October 2016 [8]. We previously supported eight VA Medical Centers (VAMCs) in implementing STRIDE using Replicating Effective Programs (REP) with external facilitation as an implementation strategy. This external facilitation involved engaging in a sequence of six scheduled calls and one in-person hospital visit by the Function QUERI (FQ) team spanning 3 months prior to program launch. Following the launch of STRIDE, hospitals participated in five additional scheduled calls with the FQ team to assess data, pinpoint obstacles to implementation, and devise strategies to overcome those obstacles. Although hospitals responded positively to support provided by the FQ team, this approach was too time-intensive (approximately 100–140 h/hospital) to be replicated on a national scale [9]. In addition, STRIDE implementation occurred without facilitation in six non-FQ hospitals, and this experience provided clear evidence that some, but not all, hospitals are able to launch EBPs solely with access to program materials and minimal technical assistance [10]. Therefore, our new protocol will evaluate strategies to monitor hospital implementation progress and only deploy higher-touch support for hospitals that have not met STRIDE program benchmarks.

We describe a protocol for a type III effectiveness-implementation hybrid trial in 32 VA hospitals to examine the impact of foundational support, consisting exclusively of low-touch activities, versus enhanced support, which begins with low-touch activities and adds “high-touch” facilitation calls for hospitals that do not meet STRIDE program benchmarks. We hypothesize that STRIDE program implementation outcomes of penetration (primary), fidelity, and adoption (secondary) will be higher at 10 (primary), 13, and 16 months at hospitals randomized to enhanced support versus foundational support. Secondary objectives are to assess the impact of effectiveness outcomes (e.g., discharge to nursing home) at implementing hospitals. We will also examine how hospitals conduct implementation in each arm (foundational and enhanced), and what baseline organizational characteristics are associated with hospitals that do not meet STRIDE program benchmarks.

### STRIDE clinical program

STRIDE’s primary objective is to optimize the physical function of older Veterans by increasing the time spent walking during hospitalization. STRIDE has four key components: (1) the program is meant to be proactive (i.e., no functional deficits required before being enrolled), (2) early enrollment is emphasized (within 24 h of admission when possible), (3) supervised ambulation to ensure safe participation in the program; and (4) hospitals have dedicated staff time to implement the program. The goal is for all Veterans who participate in STRIDE to have up to 20 min of walking within the first 24 h of admission to a general medicine ward, followed by progressive daily mobilization. Patients will be guided through their daily walking by a trained STRIDE team member, referred to as the STRIDE Mobility Assistant, who will follow established protocols. Hospitals will be advised that recommended STRIDE patients include those who are 60 years or older, admitted to a general medicine ward, do not require inpatient physical therapy, can follow one-step commands, and are able to ambulate safely. However, hospitals are allowed to set additional clinical eligibility for their program at their discretion.

### Hospital eligibility

Hospital eligibility for this study will be VAMCs that are not currently offering nor have offered a STRIDE program in the past 5 years and are willing to implement the program on a general medicine ward. Eligible hospitals will be informed of the implementation opportunity through recruitment calls, word of mouth, leveraging operational partners, and utilizing Microsoft Identity Manager (MIM) to identify potential points of contact (POCs) at hospitals. The overall goal of the project is to implement, evaluate, and sustain STRIDE at 32 VAMCs. Additional inclusion criteria include: (1) facility leadership is willing to participate in the study via signed participation agreement and (2) hospital staff agrees to attend monthly touchpoints with FQ team.

### Hospital recruitment

We will use a muti-level approach to recruit hospitals interested in implementing the STRIDE program. Starting at the national level, the FQ team will partner with the Diffusion of Excellence (DOE) to connect with Veterans Integrated Service Networks (VISN) leadership. A DOE representative will help to foster relationships through email communication and scheduling recruitment calls. Once the DOE representative has spoken with VISN level leadership to gauge interest, the FQ team will begin utilizing MIM to compile a list of hospital level leadership to engage. Utilizing this database will allow the FQ team to identify potential POCs and other interested staff at hospitals. Through email outreach, the FQ team will determine if hospitals would like to move forward with learning about the STRIDE program during 30-min virtual information sessions. Another method of recruitment will include presenting at national and VISN level monthly and quarterly calls to convey a deeper level of information about STRIDE with larger groups.

### Hospital enrollment and randomization

Hospitals will be enrolled in cohorts every 3 to 4 months with the goal of enrolling 32 hospitals. Stratified block randomization will be used to randomize hospitals 1:1 to either foundational (low-touch) or enhanced support (high-touch if needed). With the exception of statisticians, all FQ team members who have contact with hospitals will be blinded to block size [11–13]. Three baseline hospital-level variables will be used for stratification in the randomization process: facility complexity level (1a complexity vs. all others), general medicine adjusted length of stay (adjusted length of stay ≥ 4.7 days vs < 4.7 days), and whether the hospital has previously attempted to start a mobility program or received resources to start STRIDE (yes to either vs. no) [14–16]. The hospital’s complexity level will be assessed based on a VA hospital-level measure, considering the complexity of services offered, with level 1a being the most complex and level 3 being the least complex. These characteristics were chosen to represent factors likely to be associated with baseline differences in hospitals that may affect implementation outcomes — low bandwidth, presence of local innovations, competing priorities, and needs alignment [17]. General medicine-adjusted length of stay will be captured using the Strategic Analytics for Improvement and Learning Value Model (SAIL), which assesses key quality measures as well as overall efficiency at individual VAMCs [16]. Previous experience with mobility programs will be determined by standard intake forms completed by POCs at each hospital asking if (1) anyone at their facility previously tried to start a STRIDE or other mobility program on a general medicine ward in the past 5 years and (2) if they have received resources or other staffing support from their VISN or a VA program office to start a STRIDE program.

The FQ team will become aware of each hospital’s randomization arm approximately 2 weeks before the 6-month assessment period. If a hospital is randomized to the enhanced support arm, they will be informed via email and begin high-touch activities if they do not meet initial or sustainment program benchmarks, as described below. Otherwise, hospitals will receive notification that no additional steps are necessary.

### Study arms and implementation activities

REP will be used as a framework underlying all implementation support activities. While REP has been described as both an implementation framework [18] and strategy [19], in this context, we refer to REP as an implementation strategy since it consists of a collection of specific activities tailored to address identified barriers to successful implementation. It’s worth noting that REP has undergone empirical testing and validation through randomized controlled trials and has demonstrated effectiveness in promoting the adoption and fidelity of clinical interventions across various healthcare organizations, including the VA [18, 19].

**Fig. 1:**
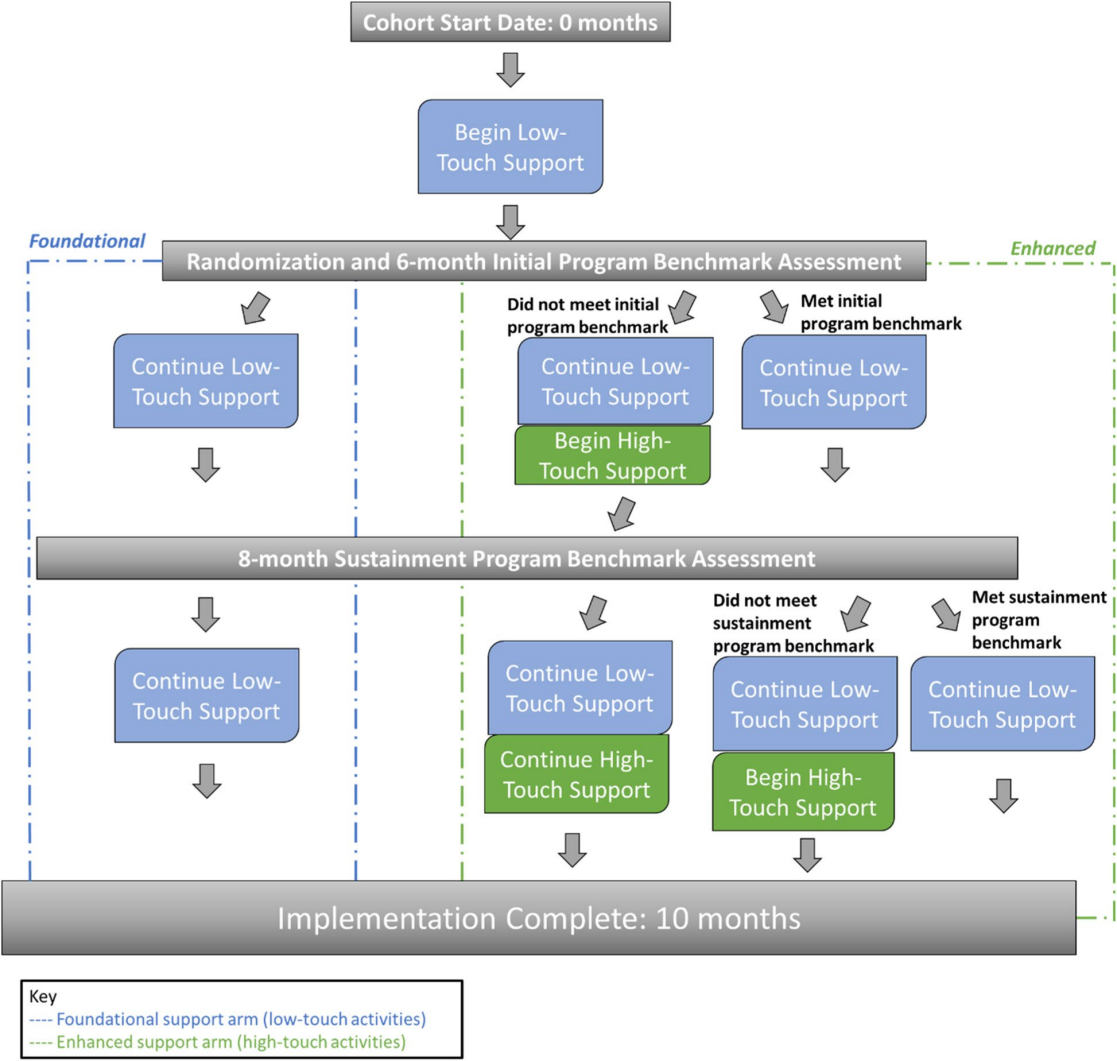
STRIDE implementation study flowchart

Figure 1 illustrates the study flow in each study arm from the start date to the completion of implementation. In line with our conceptual framework (described below) and previous STRIDE work, we will use foundational support with low-touch activities to guide the implementation of the program [18, 19]. Hospitals will begin their 10-month implementation timeline by engaging in low-touch activities for the first 6 months. At that point, all hospitals will be assessed to determine if they have successfully met the STRIDE initial program benchmark defined as ≥ 5 general medicine patients with a STRIDE walk in the Electronic Health Record (EHR) within month five or six from the start date with FQ (defined as the date of each cohort’s launch). Hospitals that have been randomized to the enhanced support arm and have not met this benchmark will begin receiving high-touch activities while those that have successfully met benchmark criteria will continue with low-touch activities only. All participating hospitals will be evaluated for the STRIDE sustainment program benchmark at 8 months defined as ≥ 10 general medicine patients with a STRIDE walk in the EHR within month seven or eight from the start date with FQ. Hospitals that have sustained their implementation will continue low-touch activities while those that have been randomized to the enhanced support arm and met the initial program benchmark at 6 months but have not sustained (did not meet the sustainment benchmark), will begin engaging in high-touch activities.

#### Low-touch activities

Based on lessons learned and input from operational partners, low-touch activities will be offered to all hospitals and will include the following five key elements that were developed and tested in our prior work. Additional detail on how low-touch activities were designed based on REP is published elsewhere [18].

1. *STRIDE toolkit:* the project will provide standardized program materials to help hospital staff implement the program, including recorded webinars.
2. *Online shared resources:* the project will provide access to pre-programmed EHR Computerized Patient Record System templates, clinical program training materials, and other standardized materials as a continually growing repository of program information.
3. *Data dashboards:* the project will provide access to a STRIDE workload report and a listing of potentially eligible patients, both hosted by VA Support Service Center, to assist hospitals with tracking their own program data.
4. *Diffusion Network:* this project network will employ a blended strategy (national and cohort level touchpoints) to support initial implementation and sustainment. It will function as a platform for gathering and disseminating local expertise, providing a collective space for peers to share best practices and exchange experiences.
  - *Welcome Call:* Hospitals will be asked to attend a “Welcome Call” at the beginning of implementation that reviews the expectations for their participation in the program as well as provides guidance on what to expect over the 10-month implementation period.
  - *Office Hours Calls:* Over a span of 10 months, hospital staff will join monthly “Office Hours” calls specific to their cohorts. During these meetings, staff will update the FQ team on their program’s growth and engage in group discussions to ask questions and tackle challenges collectively. These calls will be structured to meet the hospitals where they are in the implementation process, offering flexibility, but with a basic level of standardization.
  - *Diffusion Network Calls:* Quarterly teleconferences will be held among the national field of STRIDE hospital staff and partners. These meetings will aim to bolster implementation, offer technical guidance and continuous consultation, and showcase and exchange best practices on a national level. Examples of discussion topics will include documentation, staffing models, program success stories, research briefings, etc.
5. *Microsoft TEAMS Channels:* Hospitals will be provided with technical support through Microsoft TEAMS channels tailored to their cohorts. Hospital staff will be instructed to use these channels to pose questions, share resources, etc. In order to preserve the low-touch approach, FQ team members will be able to answer questions, but mainly count on assistance from other hospital staff for troubleshooting and providing guidance.

#### High-touch activities

Enhanced support continues with all activities provided in the foundational support arm. However, for hospitals initially randomized to the enhanced support arm that do not meet STRIDE program benchmarks at 6 (initial) or 8 (sustainment) months, respectively, they will transition to more intensive, tailored support for a period of 2 to 4 months. This will include tailored sessions designed to conduct needs assessments and address barriers through interactive problem-solving and support [20]. Each hospital will participate in a minimum of three to four synchronous sessions ranging from 30 to 60 min, with the option for additional sessions upon request. Hospitals will be asked to identify and prioritize perceived barriers to implementation, which will guide the facilitator. The facilitator, who is an implementation expert, will utilize evidence-based practices to conduct the call and guide the conversation. They will assist hospitals with problem-solving by engaging leadership when needed, providing resource materials, and identifying and addressing barriers to starting and sustaining STRIDE programs. During the sessions, additional FQ team members will be present to monitor call fidelity and implementation progress through structured notetaking. In addition, each hospital receiving high-touch activities will receive access to hospital-specific Microsoft TEAMS channels to increase accessibility to FQ team members.

## Evaluation

### Evaluation approach

**Fig. 2:**
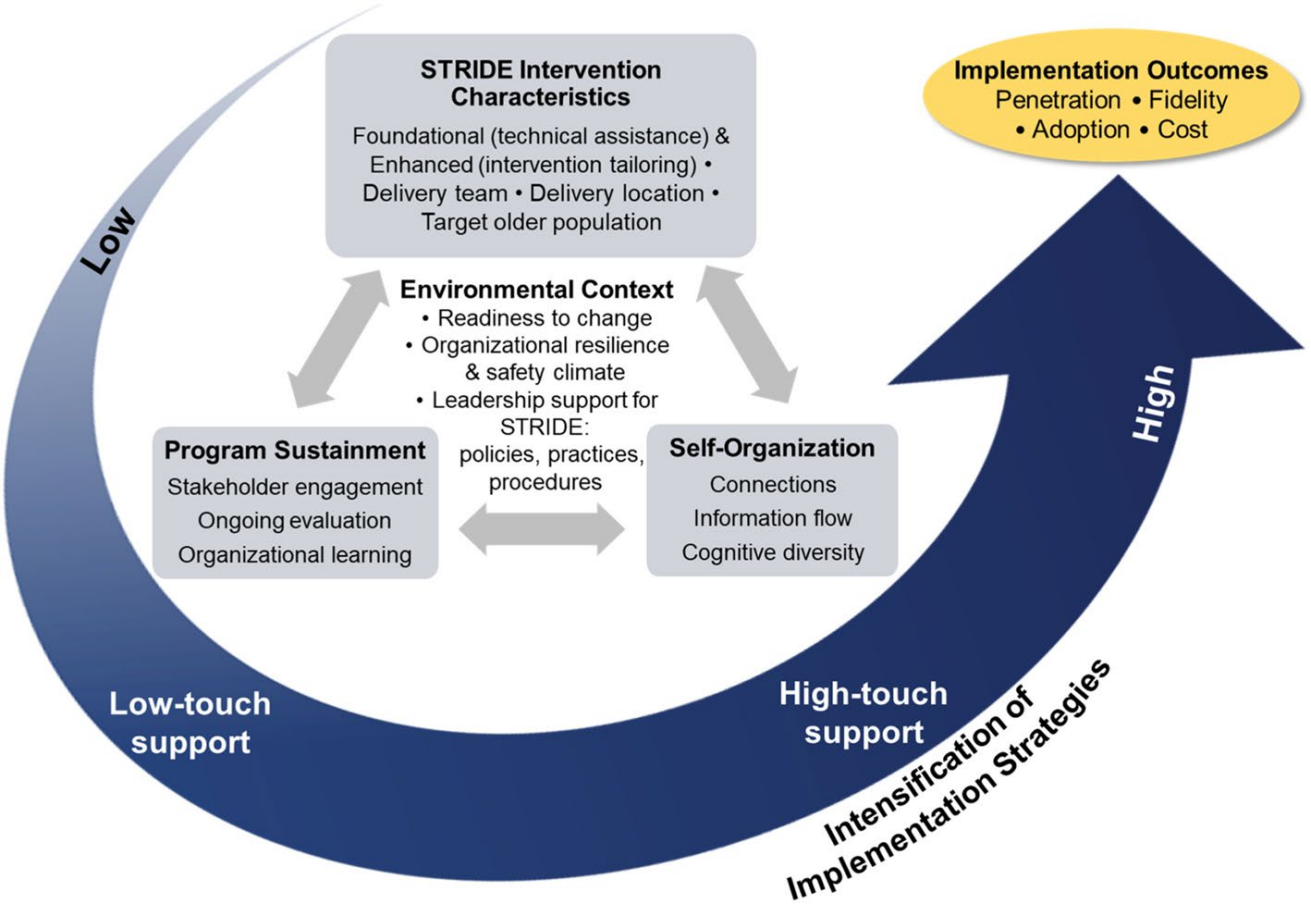
Function QUERI implementation intensification framework

We have developed a comprehensive framework (Fig. 2), adapted from Decosimo et al. [21] for evaluating implementation activities, drawing from the Dynamic Sustainability Framework [22], complexity science [23], and Proctor’s taxonomy of implementation outcomes [24]. According to our model, successful implementation of new programs relies on various factors, including the characteristics of EBPs, the environmental context of the hospital, processes that support program sustainment, and the capacity of hospitals to self-organize and solve problems effectively. Specifically, we expect that hospitals exhibiting a high readiness to change, a positive safety climate, and resilience ethos will have greater success in implementing the program [25–28]. However, due to contextual variations and organizational characteristics, we propose that providing low-touch support that encourages program adaptation to specific contexts and offers tools for ongoing evaluation will suffice for some hospitals, but not all, in successfully incorporating STRIDE into routine clinical practice. Furthermore, we suggest that consistently monitoring the progress of hospitals and implementing higher-touch support for hospitals that do not meet the benchmark criteria, directly targeting their capacity and skills for self-organization and problem-solving, will result in higher instances of program sustainment, penetration, fidelity, and value.

### Data collection/sources

The implementation of STRIDE will be assessed using an explanatory sequential mixed methods design that includes a cluster-randomized trial and qualitative data collection and analysis [29]. This will involve gathering both quantitative and qualitative data from hospital staff simultaneously to investigate contextual and experiential factors affecting hospital-level outcomes. The evaluation data (Table 1) will be collected through various mechanisms at different timepoints throughout the study and will include (1) implementation surveys, (2) semi-structured interviews with hospital staff, (3) forms noting program adaptations, and (4) detailed notes from meetings and Microsoft TEAMS. Additional data sources included VA’s Corporate Data Warehouse, which is a repository of VA EHR, and VA-purchased care data [30].

**Table 1:** STRIDE data sources

**Data source**	**Description/content**	**Collection timepoint**
Electronic Health Record	• Implementation outcomes • Effectiveness outcomes • STRIDE activity	Across study period
Implementation surveys	• Implementation and complexity science validated measures (ORIC, Organizational Resilience, PSI, etc.) • Feedback on implementation tools & resources • Barriers/challenges	0-, 6-, and 10-month intervals
Program Description Forms (Adaptations)	• Program eligibility (exclusion criteria and target population) • Staffing and equipment • Marketing and education • Documentation	0-, 6-, and 10-month intervals
Welcome Call and Monthly Office Hour Notes	• Attendance/engagement • Hospital report outs • Questions asked by hospitals • Barriers/challenges	Monthly
Quarterly Diffusion Network Calls	• Attendance/engagement • Questions asked by hospital	Quarterly
Diffusion Network Microsoft TEAMS Channel	• Questions asked by hospital	Intermittently
Enhanced Support Call Notes	• Attendance/engagement • Progress updates • Questions asked by hospitals • Facilitator’s notes • Comments from meeting chat	4-5 times throughout high-touch facilitation
Business Case Analysis	• Time spent on STRIDE planning outside time spent with Function QUERI team	3-, 6-, and 10-month intervals
Qualitative interviews	• Attendance/engagement in interview • Hospital report outs • Barriers/challenges	0-, 6-, and 10-month intervals

### Implementation outcomes

STRIDE program implementation outcomes will be assessed at the hospital level for all patient hospitalizations on general medicine wards for patients 60 years or older on admission over the specified time periods of 10 (primary), 13, and 16 months from the start date with FQ. As part of our type III effectiveness-implementation hybrid design framework, penetration (primary outcome) for a hospital is defined as the proportion of eligible hospitalizations with at least one documented STRIDE walk. Evaluating all STRIDE eligibility criteria within the EHR, such as the ability to walk at hospitalization, is not possible; therefore, with this more inclusive denominator, aiming for 100% penetration is not a feasible target. Based on preliminary data, we expect penetration to vary between 0% (indicating no program activity at the 10-month outcome assessment) and 40% (the estimated maximum possible rate based on data from existing STRIDE hospitals) [8]. Fidelity at a hospital will be assessed for eligible hospitalizations with at least one documented STRIDE walk as the percentage of eligible hospital days with “full dose” of the program, defined as two or more documented walks or one walk for more than 5 min. Program adoption at a hospital is a binary outcome defined as ≥ 5 patients with a STRIDE walk or not. The 5-patient threshold was selected to represent a minimal amount of clinical activity to suggest that the program was underway.

### Effectiveness outcomes

We will focus on hospital length of stay due to its importance to patients and implications for overall facility efficiency and performance reporting. Other important effectiveness outcomes will include inpatient fall rates and discharge to nursing home.

### Staff survey outcomes

Implementation surveys will be delivered using VA Research Electronic Data Capture (REDCap) [31] to leadership and hospital staff identified as part of the STRIDE delivery team. An initial email invitation will be sent, followed by two weekly reminders. The surveys will capture factors that are expected to influence implementation including characteristics of the intervention, team capacity to self-organize, and environmental factors. Baseline survey measures will include organizational resilience, organizational readiness, and implementation climate. The 6- and 10-month survey measures will assess program sustainability, organizational resilience, and staff experience with implementation [25–28]. Additionally, baseline and both follow-up surveys will gather information on challenges and successes to implementation based on previous FQ work [8].

### Program adaptations

Aligned with the REP framework, during implementation activities hospitals are encouraged to consider a limited number of program adaptations to enhance fit in their context without compromising fidelity. All adaptations to STRIDE delivery, planned and unplanned, will be reported using Wiltsey Stirman’s Framework for Reporting Adaptions and Modifications Expanded (FRAME), which offers a standardized approach to track modifications [32]. FRAME emphasizes reporting aspects of adaptations that are often overlooked, such as (1) the timing and manner of modifications during the implementation process, (2) whether the modification was proactive or reactive, and (3) who made the decision to make the modification. STRIDE will utilize FRAME to characterize program adaptations and assess their associations with greater implementation success. Hospitals will be asked to record adaptations at periodic times (baseline, months 6 and 10) throughout the course of the trial.

### Quantitative analysis approach

As part of our hybrid type III effectiveness-implementation study design, the primary research question compares differences in STRIDE program implementation outcomes between arms. Implementation outcomes are continuous (penetration, fidelity), and binary (adoption) hospital-level outcomes, and generalized linear models will be used to examine the effect of foundational support (low-touch) versus enhanced support (high-touch if needed) on implementation outcomes at 10 months [33]. The main predictor of interest will be an indicator variable for arm and will include indicators for the stratification variables facility complexity level (1a complexity vs. all others), whether the hospital has previously attempted to start a mobility program or received resources to start STRIDE (yes to either vs. no), and general medicine adjusted length of stay (adjusted length of stay ≥ 4.7 days vs < 4.7 days) in the model. In secondary analyses, implementation outcomes at 13 and 16 months will be assessed. We will examine how implementation outcomes change over time using descriptive methods including the 6-month time period (e.g., plots, descriptive statistics, subgroups).

We will calculate descriptive statistics for hospital-level survey measures (ORIC, Organizational Resilience, PSI, etc.), both overall and by study arm [25–28]. We will use a similar modeling approach described above to examine the effect of implementation strategy on hospital-level survey measures.

Although the primary study aim is to evaluate implementation strategies, we will also seek additional information on effectiveness with a focus on hospital length of stay and secondarily inpatient falls and discharge to nursing home. Our sample of patients for this analysis will come from hospitals that adopted the program at 10 months. The sample of patients will be from those who are hospitalized on general medicine wards and 60 years old or older at admission; some of those patients will have participated in STRIDE (i.e., treatment group) and some will have not (i.e., comparison group). Patient sociodemographic, health, and hospitalization characteristics that are potential confounders will be extracted from the EHR. We will use inverse-probability of treatment weights methods to adjust for confounding and estimate an average causal treatment effect for STRIDE.

### Business case analysis

Business Case Analysis (BCA) will assess the affordability of STRIDE for the VA [34]. We will use methods based on our previous FQ work to conduct this analysis [35]. Two types of costs will be collected at each hospital, using a standardized method to track implementation activities: (1) clinical delivery team costs, which will include personnel time and labor costs associated with preparing for and delivering the clinical programs, using micro-costing and periodic time studies. Labor costs will be based on Office of Personnel Management salary data, and durable medical equipment costs (e.g., walkers, stopwatches) will be valued at their purchase price. (2) Implementation strategy costs, including training time, training materials, and time invested by both the FQ team and hospital team members outside of program delivery time. The value will be assessed based on the implementation and quality outcomes of interest for STRIDE.

The BCA will use a decision tree to compare the expected value of costs and outcomes between arms. Additionally, one-way and probabilistic sensitivity analysis will be employed to simulate likely outcomes based on distributions informed by trial data and prior evidence for STRIDE. By modeling various plausible scenarios, the decision model will provide a practical range of estimates to communicate to hospitals and operational partners, avoiding sole reliance on statistical significance. Working with operational partners, we will establish thresholds for value [36]. The BCA will present costs by strategy and explore different assumptions, highlighting conditions required to meet VA standards for value and affordability.

### Qualitative analysis approach

The qualitative component will shed light on implementation activities and perceptions on intensification. We will sample two hospitals per cohort for qualitative interviews, regardless of cohort size, with the goal of maximizing diversity of the overall STRIDE sample by geography and implementation experience. We will conduct 30-min semi-structured interviews with hospital staff, either individually or in groups, to gain detailed insights into the facilitators and barriers affecting the implementation of EBPs. During these interviews, we will inquire about specific activities and strategies employed by their hospital to implement STRIDE [24]. Additionally, we will interview leaders who are not directly involved in the day-to-day operations (e.g., chief of staff, facility director) to gather higher-level perspectives on implementation. To analyze the data, we will use directed content analysis, incorporating a priori labels for marking high and low-touch implementation strategies based on the Expert Recommendations for Implementing Change typology [20, 37]. We will also include data-derived labels to reflect respondents’ descriptions of their experiences with barriers and implementation.

### Integration of qualitative and quantitative data

We will follow mixed methods best practices to integrate quantitative and qualitative data within the explanatory sequential mixed method design (QUAN → qual). We will combine results after separate analyses of the quantitative and qualitative data. The quantitative component will evaluate the effect of implementation strategies on implementation outcomes of interest (e.g., penetration and adoption). The qualitative component will describe implementation activities, facilitators, barriers, and staff perceptions of support intensification at each hospital. We will integrate quantitative and qualitative data using a framework matrix within the NVivo 12 Plus (QSR International) qualitative software package. In doing so, we will reveal conditions at each hospital that will help to interpret unmeasured factors that may have affected implementation outcomes.

### Sample size and power

Sample size calculations will be conducted for the STRIDE program penetration implementation outcome at 10 months. Using a two-sided *t*-test based on a sample size of 32 hospitals (randomized 1:1 to each study arm) and a type-1 error rate of 5%, we will have 80% power to detect an effect size difference of 1.0 and 90% power to detect an effect size difference of 1.2 between arms. For the primary penetration implementation outcome, assuming a standard deviation of approximately 9% based on preliminary data from ongoing work, the effect size differences that are powered to detect corresponds to a mean difference of 9% in penetration between arms for 80% power and 10.8% for 90% power.

## Discussion

### Overall goal

The mixed methods design of this study will assist in identifying the “right dose” of implementation support needed to successfully start a STRIDE program at VA hospitals with varying levels of existing resources and capacity for organizational change. It is also a crucial step in identifying the gaps between sustaining evidence-based programs after research is concluded. Clinical and administrative leaders throughout the VA Health Care System are supportive of STRIDE not only because of its potential to be an important advancement in the care of older Veterans, but also because it has proven to be a cost-saving/cost-neutral program (Kaufman B, Hastings SN, Meyer C, Stechuchak KM, Choate A, Decosimo K, et al: The business case for hospital mobility programs in the Veteran health care system: results from multi-hospital implementation of the STRIDE program, forthcoming). The VA’s commitment to spreading evidence-based programs solidifies the need for further research on the most effective and efficient strategies to disseminate and sustain these practices.

### Challenges/limitations

REP is not designed to address differences in organizational readiness or resilience (i.e., capacity), which may limit the ability of some hospitals to adopt a new EBP. Function QUERI addresses this potential limitation by supplementing foundational support activities with higher-touch activities for hospitals not meeting STRIDE program benchmarks.

## Conclusion

This work will identify the optimal implementation approach for large-scale spread of STRIDE, which could become a system-wide program aimed at tackling hospital-associated disability within the VA. In the long term, this work aims to strengthen the VA’s ability to swiftly adopt evidence-based practices through advancing the understanding of scaling proven implementation strategies for national dissemination efforts. The FQ program will contribute to building VA’s implementation capacity and workforce, leading to lasting improvements in care delivery.

## Data Availability

Not applicable.

## Abbreviations

BCA: Business Case Analysis
DOE: Diffusion of Excellence
EBP: Evidence-based program
EHR: Electronic Health Record
FRAME: Framework for Reporting Adaptions and Modifications Expanded
Function QUERI: Function and Independence Quality Enhancement Research Initiative
FQ: Function QUERI
MIM: Microsoft Identity Manager
POC: Point of Contact
REDCAP: Research Electronic Data Capture
REP: Replicating Effective Programs
SAIL: Strategic Analytics for Improvement and Learning Value Model
STRIDE: Assi*ST*ed Ea*R*ly Mob*I*lity for Hospitalize*D* V*E*terans
VA: Veterans Affairs
VAMC: VA Medical Center
VISN: Veterans Integrated Service Networks

## References

[1] Covinsky KE, Pierluissi E, Johnston CB (2011). Hospitalization-associated disability: “She was probably able to ambulate, but I’m not sure”. JAMA.

[2] Brown CJ, Redden DT, Flood KL, Allman RM (2009). The underrecognized epidemic of low mobility during hospitalization of older adults. J Am Geriatr Soc.

[3] Goodwin JS, Howrey B, Zhang DD, Kuo YF (2011). Risk of continued institutionalization after hospitalization in older adults. J Gerontol A Biol Sci Med Sci.

[4] Greysen SR, Patel MS (2018). Annals for hospitalists inpatient notes - bedrest is toxic - why mobility matters in the hospital. Ann Intern Med.

[5] Growdon ME, Shorr RI, Inouye SK (2017). The tension between promoting mobility and preventing falls in the hospital. JAMA Intern Med.

[6] Pavon JM, Fish LJ, Colón-Emeric CS (2021). Towards “mobility is medicine”: socioecological factors and hospital mobility in older adults. J Am Geriatr Soc.

[7] Brown CJ, Foley KT, Lowman JD (2016). Comparison of posthospitalization function and community mobility in hospital mobility program and usual care patients: a randomized clinical trial. JAMA Intern Med.

[8] Hastings SN, Stechuchak KM, Choate A (2023). Effects of implementation of a supervised walking program in Veterans Affairs hospitals: a stepped-wedge, cluster randomized trial. Ann Intern Med.

[9] Hastings SN, Choate AL, Mahanna EP (2018). Early mobility in the hospital: lessons learned from the STRIDE program. Geriatrics.

[10] Sperber NR, Bruening RA, Choate A (2019). Implementing a mandated program across a regional health care system: a rapid qualitative assessment to evaluate early implementation strategies. Qual Manag Health Care.

[11] Raab GM, Butcher I (2001). Balance in cluster randomized trials. Stat Med.

[12] Li F, Turner EL, Heagerty PJ, Murray DM, Vollmer WM, DeLong ER (2017). An evaluation of constrained randomization for the design and analysis of group-randomized trials with binary outcomes. Stat Med.

[13] Carter BR, Hood K (2008). Balance algorithm for cluster randomized trials. BMC Med Res Methodol.

[14] Veterans Health Administration Office of Productivity, Efficiency and Staffing (VHA OPES). VHA facility complexity model history. https://reports.vssc.med.va.gov/ReportServer/Pages/ReportViewer.aspx?/OPES/FacilityComplexity/FacilityComplexHistory&rs:Command=Render. Accessed 15 Sept 2023.

[15] Jeong IC, Healy R, Bao B (2020). Assessment of patient ambulation profiles to predict hospital readmission, discharge location, and length of stay in a cardiac surgery progressive care unit. JAMA Netw Open.

[16] US Department of Veterans Affairs. SAIL FY2022 hospital performance - all facilities. https://www.data.va.gov/dataset/SAIL-FY2022-Hospital-Performance-All-Facilities/rc4s-93qz. Accessed 7 Sept 2023.

[17] Miake-Lye I, Mak SS, Lambert-Kerzner AC (2019). Scaling beyond early adopters: a systematic review and key informant perspectives.

[18] Kilbourne AM, Neumann MS, Pincus HA, Bauer MS, Stall R (2007). Implementing evidence-based interventions in health care: application of the replicating effective programs framework. Implement Sci.

[19] Kilbourne AM, Abraham KM, Goodrich DE (2013). Cluster randomized adaptive implementation trial comparing a standard versus enhanced implementation intervention to improve uptake of an effective re-engagement program for patients with serious mental illness. Implement Sci.

[20] Powell BJ, Waltz TJ, Chinman MJ (2015). A refined compilation of implementation strategies: results from the Expert Recommendations for Implementing Change (ERIC) project. Implement Sci.

[21] Decosimo K, Drake C, Coffman CJ, Sperber NR, Tucker M, Hughes JM (2023). Implementation intensification to disseminate a skills-based caregiver training program: protocol for a type III effectiveness-implementation hybrid trial. Implement Sci Commun.

[22] Chambers DA, Glasgow RE, Stange KC (2013). The dynamic sustainability framework: addressing the paradox of sustainment amid ongoing change. Implement Sci.

[23] Pype P, Mertens F, Helewaut F, Krystallidou D (2018). Healthcare teams as complex adaptive systems: understanding team behaviour through team members’ perception of interpersonal interaction. BMC Health Serv Res.

[24] Proctor E, Silmere H, Raghavan R (2011). Outcomes for implementation research: conceptual distinctions, measurement challenges, and research agenda. Adm Policy Ment Health.

[25] Lee A, Vargo J, Seville E (2013). Developing a tool to measure and compare organizations’ resilience. Nat Hazards Rev.

[26] Helfrich CD, Li YF, Sharp ND, Sales AE (2009). Organizational readiness to change assessment (ORCA): development of an instrument based on the Promoting Action on Research in Health Services (PARIHS) framework. Implement Sci.

[27] Shea CM, Jacobs SR, Esserman DA, Bruce K, Weiner BJ (2014). Organizational readiness for implementing change: a psychometric assessment of a new measure. Implement Sci.

[28] Ehrhart MG, Aarons GA, Farahnak LR (2014). Assessing the organizational context for EBP implementation: the development and validity testing of the Implementation Climate Scale (ICS). Implement Sci.

[29] Creswell JW, Clark VLP (2017). Designing and conducting mixed methods research.

[30] (VINCI) VIaCS. VA Informatics and Computing Infrastructure. VA HSR RES 13-457, U.S. Washington D.C.: Department of Veterans Affairs; 2008.

[31] Harris PA, Taylor R, Thielke R, Payne J, Gonzalez N, Conde JG (2009). Research electronic data capture (REDCap)–a metadata-driven methodology and workflow process for providing translational research informatics support. J Biomed Inform.

[32] Wiltsey Stirman S, Baumann AA, Miller CJ (2019). The FRAME: an expanded framework for reporting adaptations and modifications to evidence-based interventions. Implement Sci.

[33] Mccullagh P, Nelder JA (1989). Generalized linear models.

[34] Sullivan SD, Mauskopf JA, Augustovski F (2014). Budget impact analysis-principles of good practice: report of the ISPOR 2012 Budget Impact Analysis Good Practice II Task Force. Value Health.

[35] Liu CF, Rubenstein LV, Kirchner JE (2009). Organizational cost of quality improvement for depression care. Health Serv Res.

[36] Leatherman S, Berwick D, Iles D (2003). The business case for quality: case studies and an analysis. Health Aff.

[37] Waltz TJ, Powell BJ, Matthieu MM (2015). Use of concept mapping to characterize relationships among implementation strategies and assess their feasibility and importance: results from the Expert Recommendations for Implementing Change (ERIC) study. Implement Sci.

